# Spatial patterns of extra-pair paternity in a waterbird colony: separating the effects of nesting density and nest site location

**DOI:** 10.1007/s00265-015-2056-0

**Published:** 2016-01-14

**Authors:** Piotr Minias, Katarzyna Wojczulanis-Jakubas, Robert Rutkowski, Krzysztof Kaczmarek, Tomasz Janiszewski

**Affiliations:** Department of Teacher Training and Biodiversity Studies, University of Łódź, Banacha 1/3, 90-237 Łódź, Poland; Department of Vertebrate Ecology and Zoology, University of Gdańsk, Wita Stwosza 59, 80-308 Gdańsk, Poland; Department of Molecular and Biometrical Techniques, Museum and Institute of Zoology PAS, 00-679 Warsaw, Poland; Medical University of Łódź, Sterlinga 1/3, 91-425 Łódź, Poland

**Keywords:** Coloniality, Great cormorant, Microsatellites, *Phalacrocorax carbo sinensis*, Sibship reconstruction

## Abstract

Centres of avian colonies are usually associated with reduced predation risk and, thus, attract individuals of high quality, while poor-quality individuals are relegated to peripheral zones. Assuming that the incidence of extra-pair paternity (EPP) is dependent on individual quality, we could expect lower incidence of extra-pair offspring in the central parts of colonies. On the other hand, central pairs often nest in higher densities, which might increase EPP rate. To test these hypotheses, we sampled 124 great cormorant *Phalacarocorax carbo sinensis* chicks from 30 broods from different zones of a colony and genotyped them at seven highly polymorphic microsatellite loci. Sibship reconstruction confirmed the presence of at least one extra-pair chick in 30.0 % of broods. We found that EPP varied significantly between the zones of the colony, with higher incidence of extra-pair broods in the peripheral zone (53.3 vs. 6.7 % of broods). Centre-edge difference in EPP was consistent with the expected distribution of pair quality and suggested that poor-quality peripheral females were more likely to solicit extra-pair interactions, possibly to gain ‘good genes’ for their offspring. By contrast, we found no evidence for density dependence in EPP rate, indicating that likelihood of raising extra-pair offspring was not constrained by limited availability of local males. The results indicate that spatial randomization of sampling within avian colonies is critical to obtain robust estimations of EPP for non-solitary species. To our knowledge, this study provides the first evidence for the centre-edge difference in EPP within a breeding colony of birds.

## Introduction

Extra-pair paternity (EPP) has been recorded in about 90 % of all bird species, demonstrating that a large majority of socially monogamous birds show varying degrees of promiscuity (Griffith et al. 2002; Neudorf 2004). Variation in EPP between major avian lineages is best explained by differences in fundamental life history traits (Møller 2000; Arnold and Owens 2002), while variation between closely related species or populations is usually explained by contemporary ecological factors (Stutchbury and Morton 1995; Petrie et al. 1998; Gohli et al. 2013). However, within populations, the frequency of solicited extra-pair copulations and resulting fertilizations may also depend on individual quality. The ‘good genes’ hypothesis assumes that females seek extra-pair copulations (EPCs) with males of higher genetic quality than their mates to gain indirect genetic benefits (viability genes or genes for attractiveness) for their offspring (Kempenaers et al. 1992; Strohbach et al. 1998). Under this scenario, poor-quality females, which usually mate assortatively with poor-quality males (Andersson et al. 1998; García-Navas et al. 2009), would be more likely to raise extra-pair offspring. As poor-quality pairs are likely to occupy low-quality nest sites (Sergio et al. 2009), we might expect more EPP in less attractive territories or nest sites, providing that high-quality males are available in the neighbourhood as potential extra-pair sires (Schlicht et al. 2015).

While some information exists on the spatial patterns of EPP in territorial birds (Bollinger and Gavin 1991; Freeman-Gallant et al. 2005; Westneat and Mays 2005), very little is known about how extra-pair offspring are distributed within colonies. In many colonial species, especially those that breed in relatively homogeneous habitats (Minias 2014), central parts of the colonies are most attractive and offer the highest benefits in terms of fitness. Centrally located nests are usually less susceptible to predation due to the following: (1) lower accessibility for most types of predators and (2) more efficient detection and deterrence of predators in the colony centre (Götmark and Andersson 1984; Yorio and Quintana 1997). For this reason, individuals of higher quality are likely to occupy safer central nest sites and individuals of lower quality are relegated to less attractive edge sites, resulting in a so-called central-periphery gradient of pair quality (Velando and Freire 2001). Central-periphery gradients in pair quality have been reported for many colonial species from diverse taxonomic groups, such as cormorants, larids, pelicans, penguins and procellarids (reviewed in Minias 2014). One of the first studies to demonstrate a central–periphery gradient found that central breeding pairs of Black-legged Kittiwakes *Rissa tridactyla* were in better physical condition and had higher fledging success than peripheral pairs (Coulson 1968). Further research on this species showed that centrally nesting individuals had higher survival rates than conspecifics occupying peripheral nest sites (Aebischer and Coulson 1990). In many colonial species, central pairs have been reported to breed earlier (Côté 2000; Gibbs et al. 2000), lay larger clutches (Montevecchi 1978; Gochfeld 1980) and raise chicks in better condition (Minias et al. 2012b). These patterns were often attributed to older birds with more experience (Blus and Keahey 1978; Pugasek and Diem 1983; Vergara and Aguirre 2006) or individuals with higher genetic quality (Minias et al. 2015a) nesting in the centre of colonies. Thus, under the assumptions of the good genes hypothesis, it might be expected that central-periphery patterns in pair quality should produce similar gradients in the incidence of extra-pair offspring and, thus, a greater proportion of extra-pair offspring in the peripheral parts of the colonies.

On the other hand, EPP rate may also be density-dependent, as both sexes are expected to have more opportunities for EPCs at higher breeding densities (Mougeot 2004). While the positive impact of local breeding densities on EPP has been demonstrated for some territorial passerines (Charmantier and Perret 2004; Stewart et al. 2010; Mayer and Pasinelli 2013), it is often debated whether EPP is related to density in most avian populations (Westneat and Sherman 1997; Tarof et al. 1998; Rätti et al. 2001). For example, it has been suggested that density effects are more likely if males control extra-pair fertilizations and females are passive targets of insemination (Dunn et al. 1994). While this may be the case in many colonial birds (Nelson 2005), there is scarce information on whether local breeding densities may affect EPP in species that breed in aggregations. Also, since central parts of the colonies are often associated with higher breeding densities (e.g., Becker 1995), the effects of central-periphery nest position and local density may easily be confounded.

The aim of this study was to investigate spatial patterns in EPP within a colony of the great cormorant *Phalacrocorax carbo sinensis*. A number of fitness-related traits follow a central-periphery gradient in great cormorant colonies, consistent with higher quality of central breeding pairs (e.g., Andrews and Day 1999; Minias et al. 2012a, b; Minias and Kaczmarek 2013). Also, unlike most colonial waterbirds with slow life histories, the great cormorant has a relatively high rate of EPP, averaging 16 % across several populations (Piertney et al. 2003). These characteristics make the great cormorant a suitable species to test hypotheses on the within-colony spatial variation in EPP. We predicted that the distribution of EPP within a colony would show the following: (1) lower incidence of extra-pair offspring in the central parts of the colony in comparison to the peripheral zone (consistent with the good genes hypothesis) and (2) higher incidence of extra-pair offspring in higher nesting densities. To test these predictions, we used a set of highly polymorphic microsatellite loci to identify extra-pair offspring and, then, conducted a spatial analysis which separated the effects of nest location and density on the rate of EPP.

## Material and methods

### Study site and species

The study was conducted in a colony of great cormorants at Jeziorsko reservoir (51° 73′ N, 18° 63′ E), central Poland, in 2010–2011. The colony was located in the area of riparian willow woodland dominated by the white willow *Salix alba* and the grey willow *Salix cinerea* in the vicinity of Proboszczowice village (51° 43.39′ N, 18° 38.61′ E) at the western shore of the reservoir. Great cormorants started to breed at Jeziorsko reservoir in 1991, when 90 nesting pairs were recorded at the site. Since then, the location and spatial organization of the colony have changed over time, and in 2010–2011, birds bred in two separate colonies at the reservoir. The colony chosen for this study was established in 2005 by ca. 40 cormorant pairs and held ca. 150 pairs in 2010–2011.

This subspecies of the great cormorant breeds colonially at a wide spectrum of inland waters throughout Europe and Asia. European populations are mostly migratory, and courtship and pair formation takes place shortly after arrival on the breeding grounds, usually in March. Breeding is relatively synchronized (peak laying in April–May in Europe), and pairs only have one brood per season. Replacement clutches may occur if the first clutch is lost but are unlikely after the loss of young (Nelson 2005).

### Nest site location and nesting density

**Fig. 1:**
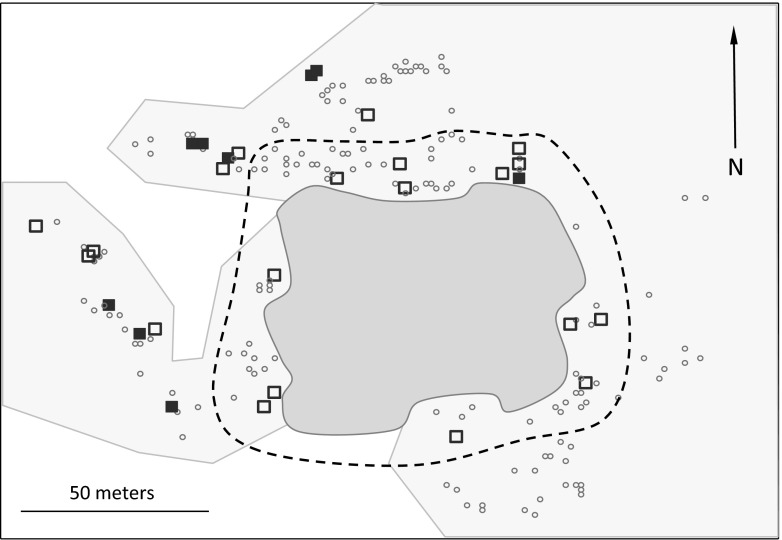
A map of the great cormorant colony at Jeziorsko reservoir, central Poland. *Dark-shaded area* marks the central pond, *light-shaded areas* mark riparian willow woodland, and the *dashed line* bounds the central zone of the colony. Nests sampled for paternity are marked with *large squares*, where *opened squares* indicate broods with no extra-pair chicks and *filled squares* indicate broods with at least one extra-pair chick. *Dots* mark active nests not sampled for paternity analyses

Each year, all active nests were mapped with a handheld Global Positioning System (GPS) unit (Garmin GpsMap 60Cx, Olathe, KS, USA) with European Geostationary Navigation Overlay Service (EGNOS) ensuring accuracy of 1–1.5 m. Collected coordinates were used to calculate distances between all the nests in the colony. On the basis of the nest-distance matrices, we calculated two measures of nesting density: (1) small-scale nesting density (nest density within a radius of 5 m of each nest) and (2) large-scale nesting density (nest density within a radius of 15 m of each nest). All the nests were also assigned to either the central or peripheral zone of the colony. Since the shape of the colony was irregular and all the pairs clustered around a central open-water pond, the nests located within a distance of 20 m from the pond were considered central, while all other nests were considered peripheral (Fig. 1). Using a threshold distance of 20 m allowed us to obtain a roughly equal division of nests between the zones, with 43.6 and 46.0 % of all nests assigned to the central zone in 2010 and 2011, respectively.

### Field procedures

The colony was visited from mid-March, when first pairs initiated laying. Laying dates and clutch sizes were recorded for 50 broods per year. Laying was relatively synchronized, and the large majority of clutches were laid by the end of April in both years. For the purpose of paternity analyses, we randomly selected at hatching 30 broods with 124 offspring (14 broods in 2010, 16 broods in 2011; all hatched between 14 April and 09 May). Only broods where all eggs hatched successfully were selected. Broods were equally distributed among the central and peripheral zones of the colony (15 broods per zone), and the mean size of the sampled broods was 4.13 ± 0.11 [SE] (range 3–6) offspring. Within each zone, we sampled broods from a wide spectrum of nesting densities (large-scale nesting density range 1–15 vs. 1–13 nests/15-m radius in the central and peripheral zone, respectively), which allowed us to separate the effects of nesting density and central-periphery nest location on EPP rate. As a result of this sampling design, our set of selected broods showed no significant differences in an average nesting density between the central and peripheral zones of the colony (small-scale nesting density 1.53 ± 0.50 vs. 1.60 ± 0.35 nests/5-m radius, *t* = 0.11, *df* = 28, *p* = 0.91; large-scale nesting density 5.53 ± 1.08 vs. 6.73 ± 1.04 nests/15-m radius, *t* = 0.80, *df* = 28, *p* = 0.43). Peripheral nests from the south-eastern part of the colony were excluded from sampling (Fig. 1), as they were largely inaccessible to researchers. To minimize the problem of non-independence, we avoided selecting the same nest sites in both seasons. Great cormorants have high nest site fidelity (Schjørring et al. 2000), so this likely excluded repeated measurements of the same pairs. Blood samples (ca. 10 μl) were collected soon after hatching by puncturing the ulnar vein of nestlings. The samples were immediately suspended in 96 % ethanol and stored until laboratory analyses.

### Genetic analysis

For genotyping, we used seven microsatellite loci previously developed for the great cormorant (Piertney et al. 1998). Forward primers were labelled with fluorescent dyes (D2, D3, D4, Sigma-Aldrich, Poland). We used a multiplex PCR Kit (Qiagen) in 15-μL total volume to simultaneously amplify five loci (PcD 4, PcD 6, PcT 1, PcT 3, PcT 4). A separate PCR was used to amplify the two other loci (PcD 2, PcD 5) using Polimerease mix (Sigma-Aldrich) in 25-μL total volume. We used a 55 °C annealing for both types of reactions. We genotyped the PCR products using a Beckman Coulter CEQ 8000 capillary automated sequencer at the Museum and Institute of Zoology, Polish Academy of Science (Warsaw, Poland). We scored alleles visually using the Beckman Coulter Fragment Analysis Software. The mean number of alleles per locus was 26.3 (10–53 alleles), and observed heterozygosity ranged from 0.57 to 1.00 (details for larger dataset in Minias et al. 2015b). The combined non-exclusion probability for sib identity was 4.17 × 10^−4^, as calculated in Cervus 3.0.3 (Kalinowski et al. 2007).

### Estimating EPP

We did not collect DNA from parents, so to determine the number of extra-pair young, we used two methods of sibship reconstruction among the nestlings. Firstly, we used a maximum likelihood method implemented in the program COLONY v2.0 (Wang 2004) to partition nestlings into full- and half-sib clusters. Maximum likelihood partitioning is considered more powerful than the pairwise approach because more information on entire families rather than just pairs of individuals is extracted and utilized (Wang and Santure 2009). In the analysis, the error rate of genotyping was set to 0.025 as suggested by Wang (2004). We did not allow for multiple maternity within broods, as despite a large body of molecular paternity studies in *Phalacrocorax* genus and other genera from Suliformes order (Graves et al. 1993; Baumgarten et al. 2001; Dearborn et al. 2001; Anderson and Boag 2006; Baião and Parker 2009; Calderón et al. 2012), no sound empirical evidence was found for either conspecific brood parasitism or quasi parasitism in this group of birds (reviewed in Yom-Tov 2001; Griffith et al. 2004). Secondly, we tested for full-sibling relationships using likelihood ratio tests based on Queller and Goodnight’s *r* (Queller and Goodnight 1989; Goodnight and Queller 1999) implemented in KINGROUP v2 (Konovalov et al. 2004). To identify extra-pair chicks within broods, a primary hypothesis of full siblings was tested against a null hypothesis of half siblings. Ten thousand simulations were carried out to assess the significance of likelihood ratios (*p* < 0.05). Only the chicks for which the results of both methods matched (indicated half-sib relationships with nest mates) were considered to result from extra-pair fertilizations.

### Statistical analyses

We used a generalized linear model with a binomial distribution and logit link function to test for the effects of nest site location (binary response: central vs. peripheral zone) and nesting density on the occurrence of extra pair paternity. Broods were used as the unit of the analysis, and binary codes were assigned for each response category of the dependent variable (0—no extra-pair offspring, 1—at least one extra-pair offspring). Small- and large-scale nesting densities were log-transformed prior to the analysis to improve normality. Both measures of density showed only moderate correlation (*r* = 0.48, *n* = 30, *p* = 0.007) and, thus, were included as covariates into one model without violating the assumption of little/no multicollinearity (|*r*| < 0.8) in the data. The effect of year was entered as a fixed factor, and laying date was entered as a covariate in the models. Stepwise procedures of backward removal were used to select for significant independent variables, and their significance was determined using the Wald *χ*^2^ statistic. All statistical analyses were performed with STATISTICA 10.0 (StatSoft, Tulsa, OK, USA).

## Results

Based on the sibship reconstruction, 30.0 % of broods were classified as containing at least one extra-pair offspring (*n* = 30), and in total, 10.5 % of chicks were identified as resulting from extra-pair fertilizations (*n* = 124). In five out of nine extra-pair broods, only one chick per brood was classified as extra-pair, while in the other four broods, two chicks per brood were classified as extra-pair.

**Fig. 2:**
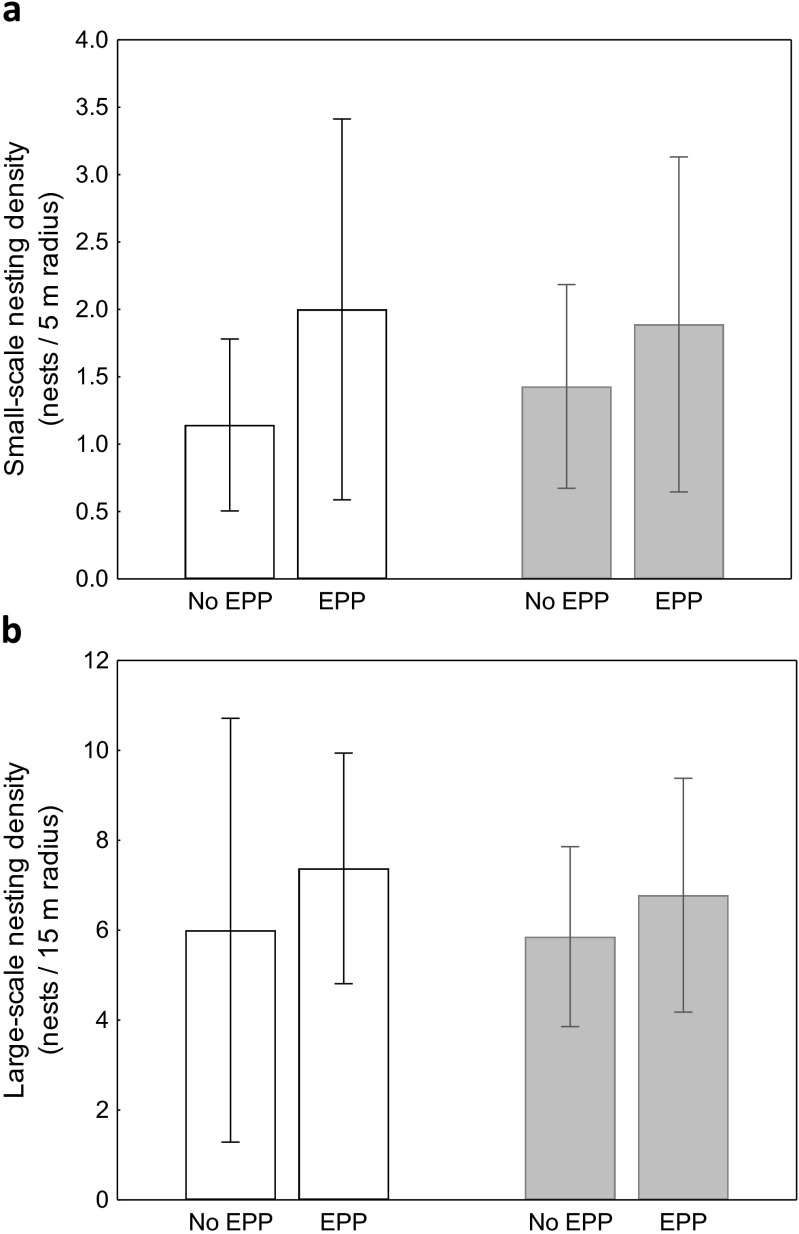
Nesting density of broods with no extra-pair chicks (no EPP) and with extra-pair chicks (EPP) in the colony of the great cormorant; shown separately for the peripheral zone of the colony (*white bars*) and for the entire colony (*shaded bars*) at small- (**a**) and large-scale (**b**) nesting densities. Means ± 95 % confidence intervals are shown

The occurrence of EPP differed significantly between the zones of the colony, and there was a higher incidence of extra-pair broods in the peripheral compared to the central zone (53.3 vs. 6.7 %; Wald *χ*^2^ = 5.60, *df* = 1, *p* = 0.018; Fig. 1). By contrast, the EPP rate did not vary with nesting density. Neither small- nor large-scale nesting density had significant effects on the occurrence of EPP (small-scale density: Wald *χ*^2^ = 0.39, *df* = 1, *p* = 0.53; large-scale density: Wald *χ*^2^ = 0.66, *df* = 1, *p* = 0.42; Fig. 2). Consistently, we found no effect of nesting density on the occurrence of EPP when the analysis was restricted to the peripheral zone, where nearly 90 % of extra-pair broods were recorded (small-scale density: Wald *χ*^2^ = 0.07, *df* = 1, *p* = 0.79; large-scale density: Wald *χ*^2^ = 1.57, *df* = 1, *p* = 0.21; Fig. 2). Occurrence of EPP did not differ between years (Wald *χ*^2^ = 0.95, *df* = 1, *p* = 0.33) and did not vary with laying date (Wald *χ*^2^ = 0.00, *df* = 1, *p* = 0.99).

## Discussion

In this study, we confirmed the occurrence of the centre-edge difference in EPP within a colony of great cormorants. Most of the broods with extra-pair offspring were recorded in the peripheral part of the colony, while incidence of EPP in the central zone was negligible. By contrast, we found no effect of density on the level of EPP.

Although estimates of EPP are available for many colonial waterbirds, spatial analyses of EPP distribution within colonies are scarce, and we are aware of no study that tested for the central-periphery pattern of EPP distribution within a breeding colony of birds. A recent study of the colonial blue-footed booby *Sula nebouxii* showed that the spatial variation of EPP across a colony was determined by habitat structure (number of obstacles to locomotion) and was quadratically related to breeding density (Ramos et al. 2014). It was proposed that individuals nesting at intermediate density were most likely to be accessed by males foraying for EPCs, while obstacles in the vicinity of a female’s nest constrained the access of foraying males (Ramos et al. 2014). These patterns have been attributed to habitat complexity (forest floor with embedded boulders and remains of dead trees) that restricted birds’ movements throughout the colony.

While the centre-edge difference in EPP has not been tested in the blue-footed booby, there was some indication for the good genes hypothesis in this species, as extra-pair courtship was more common in females with younger mates (Kiere and Drummond 2014). Strong support for the good genes hypothesis was also found for the European shag *Phalacrocorax aristotelis*. It has been shown that the reproductive success of females is correlated negatively with the number of solicited EPCs and that the most successfully reproducing males attracted more females to their territories for EPCs (Graves et al. 1993). Although we had no direct information on the quality of adult birds in our colony, there is relatively strong empirical evidence for the poorer quality of peripherally nesting pairs. Previous studies in the same colony indicated that peripheral pairs started breeding later and raised fewer fledglings than central pairs (Minias et al. 2012a; Minias and Kaczmarek 2013). Peripheral pairs were also younger (Minias 2012), and they invested more in female offspring, which are smaller and, thus, less expensive to rear (Minias et al. 2014b). Taking all of these previous results into account, we suggest that the centre-edge difference in EPP rate is due to poor-quality peripheral females seeking genetic benefits for their offspring via extra-pair matings.

We also found that the EPP rate in the great cormorant was not related to local nesting density. This may suggest that females do not engage in EPCs with closest neighbours but seek interactions with males from more distant, possibly central, parts of the colony. A similar mechanism has been described in the blue-footed booby, where extra-pair chicks were not sired by neighbours (Ramos et al. 2014). Spatial patterns of EPP in other colonial birds seem to support a general lack of density effects, although there is limited information available. No relationship between local nesting density and individual numbers of EPCs has been reported for a colonial white ibis *Eudocimus albus* (Frederick 1987), and the number of extra-pair fertilizations was not related to nesting density in a mixed colony of Ross’s geese *Chen rossi* and lesser snow geese *Chen caerulescens* (Dunn et al. 1999). Lack of density effects in geese has been attributed to the ability of females to resist EPCs and control the success of extra-pair fertilizations. Under this mechanism, greater density may not increase the benefits of EPP to females, because adding more potential mates will not necessarily increase opportunities for extra-pair matings, at least above a minimum level of density (Dunn et al. 1999). We hypothesize that a similar mechanism may explain the lack of relationship between EPP and nesting density in the great cormorant.

Our estimation of EPP rate in the great cormorant (ca. 10 % of offspring) is among the highest ever recorded in colonial waterbirds. More extra-pair offspring per brood have been reported only in two species, the waved albatross *Phoebastria irrorata* (Huyvaert et al. 2000) and the black-headed gull *Chroicocephalus ridibundus* (Ležalová-Piálková 2011). High EPP rate in the great cormorant is surprising, as this species is characterized by relatively slow life history. Adult survival rate was estimated at 0.88–0.90 for the *Ph. c. sinensis* subspecies (Frederiksen and Bregnballe 2000; Hénaux et al. 2007), which translates into an average lifespan of 6 years (Schjørring et al. 2000). It is predicted that in such long-lived species, abandonment of a reproductive event is likely to be adaptive under uncertainty of paternity (Mauck et al. 1999), thus, constraining the evolution of a high EPP rate. Nevertheless, some other life history traits of the great cormorant do not clearly fit into the pattern of slow life history. Most importantly, great cormorants have relatively high fecundity driven primarily by a large clutch size (on average, three to four eggs), which distinguishes this species from most seabirds (Bregnballe 2006). As large clutch size decreases the risk of retaliation via divorce, it could possibly facilitate the occurrence of elevated EPP rate (Arnold and Owens 2002).

To our knowledge, this study provides the first evidence for the centre-edge difference in EPP within a breeding aggregation of birds. The spatial distribution of EPP was consistent with the expected distribution of pair quality within the colony and suggested that poor-quality peripheral females were more likely to solicit extra-pair interactions to gain good genes for their offspring. The results may have important practical consequences for further research on EPP in colonial species, indicating that spatial randomization of brood sampling within the colonies is critical to obtain robust estimations of EPP.
